# Physics Comes to the Aid of Medicine—Clinically-Relevant Microorganisms through the Eyes of Atomic Force Microscope

**DOI:** 10.3390/pathogens9110969

**Published:** 2020-11-20

**Authors:** Mateusz Cieśluk, Piotr Deptuła, Ewelina Piktel, Krzysztof Fiedoruk, Łukasz Suprewicz, Paulina Paprocka, Patrycja Kot, Katarzyna Pogoda, Robert Bucki

**Affiliations:** 1 Department of Medical Microbiology and Nanobiomedical Engineering, Medical University of Bialystok, PL-15222 Bialystok, Poland; mticv1@gmail.com (M.C.); piotr.deptula@umb.edu.pl (P.D.); ewelina.piktel@wp.pl (E.P.); krzysztof.fiedoruk@umb.edu.pl (K.F.); lukaszsuprewicz@gmail.com (Ł.S.); 2 Department of Microbiology and Immunology, Institute of Medical Science, Collegium Medicum, Jan Kochanowski University in Kielce, PL-25317 Kielce, Poland; paulina.paprocka@ujk.edu.pl (P.P.); patrycja.kot@ujk.edu.pl (P.K.); 3 Institute of Nuclear Physics Polish Academy of Sciences, PL-31342 Krakow, Poland; katarzyna.pogoda@ifj.edu.pl

**Keywords:** AFM, microbiology, bacteria, biofilm, fungi, viruses, antimicrobial therapies

## Abstract

Despite the hope that was raised with the implementation of antibiotics to the treatment of infections in medical practice, the initial enthusiasm has substantially faded due to increasing drug resistance in pathogenic microorganisms. Therefore, there is a need for novel analytical and diagnostic methods in order to extend our knowledge regarding the mode of action of the conventional and novel antimicrobial agents from a perspective of single microbial cells as well as their communities growing in infected sites, i.e., biofilms. In recent years, atomic force microscopy (AFM) has been mostly used to study different aspects of the pathophysiology of noninfectious conditions with attempts to characterize morphological and rheological properties of tissues, individual mammalian cells as well as their organelles and extracellular matrix, and cells’ mechanical changes upon exposure to different stimuli. At the same time, an ever-growing number of studies have demonstrated AFM as a valuable approach in studying microorganisms in regard to changes in their morphology and nanomechanical properties, e.g., stiffness in response to antimicrobial treatment or interaction with a substrate as well as the mechanisms behind their virulence. This review summarizes recent developments and the authors’ point of view on AFM-based evaluation of microorganisms’ response to applied antimicrobial treatment within a group of selected bacteria, fungi, and viruses. The AFM potential in development of modern diagnostic and therapeutic methods for combating of infections caused by drug-resistant bacterial strains is also discussed.

## 1. Introduction

### Application of Atomic Force Microscopy (AFM) in the Field of Microbiology

A set of specialized equipment and experimental methods that are currently used to develop new antibiotics and antiviral compounds includes, among others, such techniques as flow cytometry [1], spectroscopy [2], fluorometry [3], scanning electron microscopy (SEM) [4], and transmission electron microscopy (TEM) [5]. Recently, atomic force microscopy (AFM) found its place as a technology with great potential to study the microorganisms themselves as well as the molecules that might control their growth. Historically, in 1986 Binnig, Quate, and Gerber invented atomic force microscope to image nonconductive surfaces with atomic resolution [6,7,8]. From that time, AFM has been employed in a large spectrum of disciplines such as solid state physics, molecular engineering, semiconductor technology, surface chemistry, polymer science, and medicine [9,10,11], becoming one of the best tools in terms of efficiency and flexibility to probe materials and biological samples [12,13]. 

**Figure 1:**
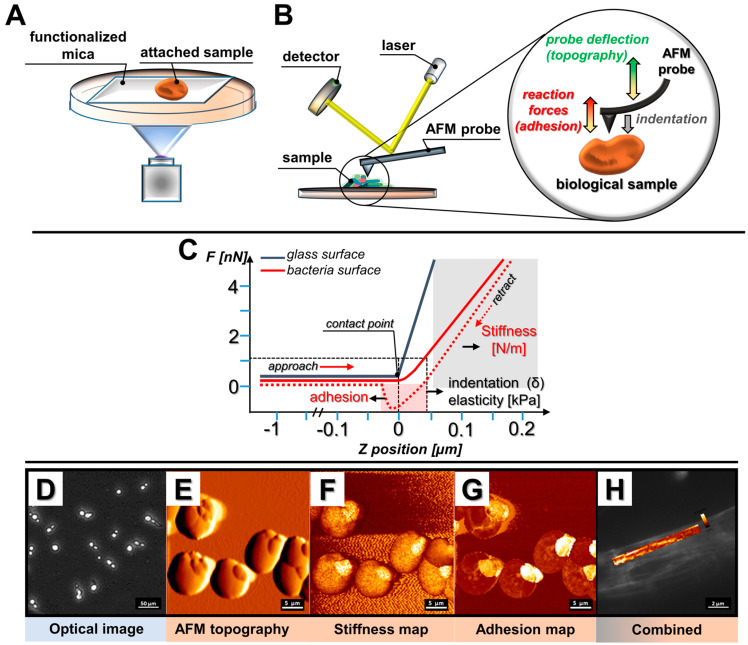
Examples of main atomic force microscopy (AFM) applications in microbiological research. (**A**) Schematic representation of sample immobilization; (**B**) schematic representation of AFM measurements; (**C**) force vs. displacement curve registered when force is applied to reference (glass surface) or investigated (bacteria) sample; (**D**) optical image of the sample; (**E**) sample topography; (**F**) stiffness mapping; (**G**) adhesion mapping; (**H**) combined fluorescence imaging and stiffness mapping.

Over the years, different AFM working modes have been developed, permitting the investigation of samples with varied size, adhesion, stiffness, and architecture features. The atomic force microscope in its basic application provides two imaging modes, known as static mode and dynamic mode, to collect the data from the sample’s surface. Nevertheless, apart from the standard modes, a broad spectrum of new modes was developed in order to control probe–sample interactions and record mechanical properties of samples without damaging them, with the quantitative imaging (QI™) mode of JPK Instruments™, the peak force™ tapping mode of Bruker™ or the “jumping mode” of Nanotec™ as the most well-recognized. Forces used in these modes can be as low as on the order of pN and with that users can achieve high resolution imaging with quantitative property mapping of mechanical and morphological features. Typically, analyses are carried out using a cantilever with a sharp, pyramidal silicon nitride tip that contacts the surface of the immobilized sample during imaging, though numerous cantilever tip shapes, including conical, sphere, or even tipples, might be employed for the purpose of AFM research (Figure 1A) [6]. The cantilever acts as a probe scanning a planar substrate, providing its topographic map. When the tip scans the sample in the *x* and *y* directions to obtain the images, the soft cantilever bends when it comes in contact with its surface in the *z* direction. As it bends, the deflection is detected by the movement of a laser beam reflected from the tip [8,14] (Figure 1B). That deflection is recorded and converted to forces using the spring constant of the cantilever in the software. While the probe scans the substrate, the adhesion forces between the sample and tip, and the stiffness of the sample are being recorded [15]. Adhesion is measured from the retraction curve, while stiffness is from the approach curve (Figure 1C). Additionally, while using AFM with a camera, the user can capture optical images (Figure 1D), topography (Figure 1E), stiffness map (Figure 1F), adhesion map (Figure 1G), and combine images, e.g., the overlay of fluorescence and stiffness (Figure 1H). A detailed description of most AFM modes along with their modifications was reviewed recently by Toca–Herrera [16].

An ever-growing number of studies has confirmed that AFM-based experiments provide an opportunity to gain biomechanical parameters at nanoscale resolution to characterize the effects of antimicrobial agents on bacteria, fungi, and viruses, as well as to investigate microorganisms’ interaction with host cells during inflammatory response occurring at the site of infections [4,17,18,19,20]. One of crucial advantages of AFM over other microscopy-based methods is the possibility to perform analyses in experimental conditions closely mimicking the physiological environment of living microorganisms. Notably, AFM allows the researcher to set out the experiment in air, vacuum, and, most importantly, in water containing media dedicated for biological applications. Therefore, AFM-based analysis allows for imaging of time-dependent processes without drying of samples, thus resulting in less fixing-associated artifacts [15,21]. In comparison, the well-recognized and broadly utilized SEM technique can only test the frozen or fixed samples, providing information before and after the treatment; however, it does not show what happened during the course of treatment in real time. In effect, AFM has become one of the best tools for real-time observations [22,23]. Moreover, AFM offers higher imaging resolution when compared to other microscopic methods. For instance, in SEM there is a possibility to observe images in a resolution of 1 nm, while AFM can produce images with 0.1 nm resolution [24,25]. Indeed, AFM allows recording parameters describing morphological changes of the object as nanotopography [26,27,28]. Additionally, interactions between the cantilever and tested object permit the delivery of information about such phenomena as stiffness, adhesion, and friction [29,30]. 

**Figure 2:**
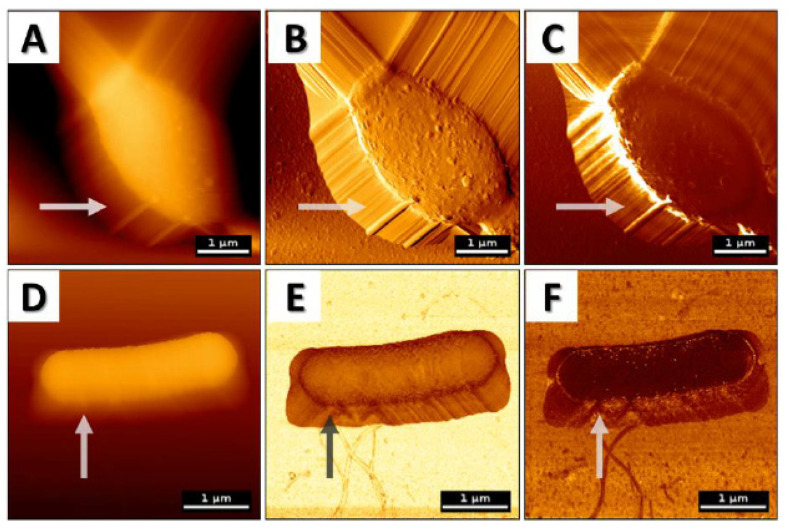
AFM topography images of (**A**–**C**) *Candida albicans* measured in contact mode (probe is in physical contact with the surface and scans the sample horizontally) and (**D**–**F**) *Bacillus subtilis* in quantitative mode (QI) (force-distance curves are recorded at every pixel of the image, meaning that the probe moves vertically towards the surface) with artifacts due to cantilever slippage and sample movement. (**A**) Topography image; (**B**) error signal; (**C**) lateral deflection that corresponds to friction forces; (**D**) topography image; (**E**) stiffness map acquired from the slope of the force-distance curve while approaching and indenting the surface; (**F**) adhesion map acquired from the force-distance curve when retracting from the surface. Arrows present artifacts recorded using AFM.

Nevertheless, in order to properly collect and then analyze the data obtained during AFM analysis, one should be aware of the limitations and difficulties associated with this technique. Firstly, it is important to note that when acquiring AFM images, users can observe artifacts due to an improper choice of cantilever tip or scanner movements (Figure 2), and some of these may lead to misleading analyses and/or incorrect conclusions [31,32,33]. Among the other factors responsible for such artifacts’ generation in AFM images, (i) thermal drift caused by temperature variations [31,34], (ii) deformation and damage due to probing in contact [32], (iii) environmental instability such as sample movement [31,33], or (iv) even software side settings [31,32,35] are well recognized.

Secondly, for the purpose of precise location of microbial cells during scanning and carrying out a reliable examination in strictly defined areas of the cell, it is necessary to immobilize a microorganism on the probed surface. Such manipulation prevents the creation of artifacts and/or damage of AFM tip during the analysis. Importantly, the immobilization step should not affect the integrity of the microorganism surface in a chemical or structural way. Most bacteria do not adhere well to solid surfaces or glass; thus, different techniques are required for proper bacterial cell immobilization, including (i) drying of the sample, (ii) passing suspension through the filters to stock the cells into the pores [36], (iii) soft gels [37], or (iv) surface coatings that assure charge drawing immobilization [36,38]. If there is a need for high-resolution imaging, it is advantageous to employ mica surface characterized by good adsorption and low background noise [39]. Notably, although in most basic biofilm-focused studies bacteria are placed directly onto glass, allowing for cells to adhere and to form biofilm communities, occasionally bacteria adhere relatively weakly; thus, AFM probes can easily detach cells during scanning. In such cases, mechanical entrapment in porous filters and soft gels like agar or agarose provides a reliable option for immobilization [36,40,41,42,43]. Noteworthy, while performing in-air scanning, this technique enhances the contrast of images [37]. Moreover, surface coatings such as polyethyleneimine (PEI), polydopamine (PDA), poly-l-lysine (PLL), poly-D-lysine (PDL), and aminosilanes (like APTES) allow for more consistent and controlled adhesion processes [36,44,45,46,47]. Nevertheless, in such an approach, interactions between the coating agents and microorganisms have to be taken into consideration. Although PLL is widely used as one of the surface coatings, its antimicrobial activity is a well-recognized feature, which may influence the viability of bacterial cells and thus induce changes in AFM measurements and mapping [48]. In addition to these immobilization approaches, surface coating can be used directly on AFM cantilever for single-cell force spectroscopy (SCFS) to mount bacteria onto tipless or colloidal cantilevers for specific adhesion/binding measurements without destruction of the cells [44,45].

It is also noteworthy that during the planning of the experiment, additional limitations, i.e., magnification gap between microscopes, should be taken under the consideration. For instance, although a prior AFM scan of the sample is visualized and localized using optical microscopy, a considerable difference in the magnification range of AFM and optical microscope makes it difficult to relocate the same sample area once the AFM probe is withdrawn from the surface. Such manipulation considerably hampers the correlative microscopic observations using AFM and other microscope techniques, such as fluorescence. For this reason, improved relocation methods, which are highly adjusted to specific experimental conditions and allow for repeated tip-sample relocation of micro- and nanosized samples for AFM imaging, are constantly developed. One of the newest achievements in this field was published recently by Abu Quba et al. [49]. As the authors demonstrated, the use of commercially available TEM grids with particles and cells fixed on the top permits a fast and cost-effective localization of nano region-of-interest and implementation of AFM/ESEM (environmental scanning electron microscopy) correlative microscopy analyses. Importantly, such an approach helps to detect AFM artifacts without any adaptations of AFM [49]. 

Finally, factors such as the geometry of the probe or the strength of imaging can significantly reduce the resolution of images and thus result in a collection of incorrect data, causing displacement of the examined object or even its damage. In order to limit potential structural damage of the tested cells and to maintain adequate image resolution at the same time, it is crucial that the forces between the probe and the tested sample are controllable. Some reports suggest that decreasing this attraction force is possible by performing the measurement in an aqueous environment rather than imaging in the air. At the same time, it is recommended that AFM-based analysis of any new biological sample should be preceded by investigation of the effect of the pH and ion concentration in a used buffer to adjust applied force during analysis acquisition [50]. 

## 2. AFM-Based Investigation as a Novel Approach to Fight with Drug Resistance in Bacteria and Fungi

### 2.1. Resistance to Antibiotics—An Emerging Problem in Medical and Environmental Microbiology

Since their pioneering first application in 1940s, antibiotics are continuously used to treat patients with bacterial infections. Over the years these “magic bullets” have significantly reduced the number of deaths, but their misusing and overusing has resulted in adaptation of microorganisms to selective pressure exerted by them by means of developing different molecular mechanisms of resistance [51]. According to the Centers for Disease Control and Prevention (CDC), each year in the United States at least 2 million people become infected with drug-resistant bacteria and at least 23,000 people die as a result of these infections [52]. Moreover, the World Health Organization (WHO) predicts that drug-resistant diseases could cause 10 million deaths each year by 2050 [53]. Expanding drug resistance is observed in the course of infections such as those caused by gram-positive cocci, e.g., methicillin-resistant *Staphylococcus aureus* (MRSA), and gram-negative rods, e.g., multidrug-resistant (MDR) *Klebsiella pneumoniae,* and extensively drug-resistant bacteria (XDR) such as carbapenemase-producing *K. pneumoniae* or *Mycobacterium tuberculosis* [54,55,56,57,58,59]. Therefore, the search for novel and innovative analytical and diagnostic methods that will facilitate characterization and elimination of those antibiotic-resistant bacteria is of great significance and is challenging at the same time.

In this field of research, the most widespread application of atomic force microscopy includes (i) the detection of changes in microbes’ morphology or abnormalities in their structure upon antibiotic-induced killing, thus, indirectly measuring the microbe susceptibility profile, (ii) analysis of the nanomechanical changes in the microbial cell envelope, e.g., stiffness, in order to understand mechanisms associated with drug resistance as well as (iii) the investigation of colonization and adhesion mechanisms of microbial cells, which is crucial for the biofilm-forming ability of most pathogens [17,60]. 

### 2.2. Analysis of Nanotopography of Pathogens as an Approach to Elucidate Viability of Microbes and Antibiotics’ Mechanism of Action

#### 2.2.1. Bacteria

One of the most common applications of AFM in microbiology research is the direct visualization of microorganisms‘ morphology upon exposure to antibiotics, and one of the first was performed by Butt et al. on dry archaebacterium (*Halobacterium halobium*) [61]. Briefly, the observation and quantification of alterations in the bacterial cell envelope architecture exposed to antibiotics may provide valuable information on the potential mechanism of action of the tested agents.

Atomic force microscopy is particularly useful for the determination of the activity of agents with membranolytic mechanism of action, and understanding the consequences of their insertion into bacterial membrane. For such purpose, Meincken et al. employed AFM to investigate the mechanisms of magainin 2 (Mag 2a), PGLa (peptidyl-glycylleucine-carboxyamide), and melittin-induced damage of *E. coli* cell envelope [28]. The AFM study revealed that the effects of these three peptides include the increase in surface roughness and lesions in the cell wall with the higher effect observed for Mag 2a and non-selective melittin when compared to PGLa. A thorough AFM-based analysis allowed us to conclude that changes in surface roughness were induced by peptide integration into the outer membrane, resulting in a “crumpling” effect because of the increased surface area. This study also confirmed the previous results reporting the magainin effect on bacterial cell membranes [62]. AFM analyses were also a basis for the conclusion that the destruction and reorganization of lipid arrangement within the outer membrane supports peptide translocation and insertion into the sensitive inner membrane that lead to cell lysis and death. The authors observed that all tested peptides caused significant damage to the apical surface of the bacterial cell wall and that the above is most likely the consequence of the inner membrane damage rather than the outer one. Simultaneously, the atomic force microscope was presented as a powerful tool to distinguish between the types of peptide-induced damage of bacteria. While melittin causes big gashes in the cell envelope and noticeable leakage of cytosolic fluid, indicating the damage to the inner membrane, Mag 2a was reported to cause pronounced vesiculation of the outer membrane [28]. Most recently, Overton et al. extended the knowledge on Mag 2a-induced killing and investigated the biophysical consequences of magainin 2 treatment using AFM combined with fluorescence observations [63]. The authors demonstrated that upon treatment, *E. coli* cells are able to maintain the stable cellular turgor pressure, despite the permeabilization of cellular membranes by Mag 2a, which is governed by the bacterial homeostasis machinery. Further exposition of bacteria to magainin 2 treatment eventually leads to a decrease of turgor pressure accompanied by remodeling of the outer membrane, resulting in its increased elasticity and greater adhesion properties. This provided new insights into the resistance of some gram-negative bacteria to the membrane-destructive effects of antimicrobial peptides [63].

In another study, Domingues et al. reported surface changes of *E. coli* and *S. aureus* after the addition of antimicrobial protein rBPI_21_ using AFM as a tool to visualize the surface disturbance accompanied by cell lysis [64]. AFM was also employed to characterize the polymyxin B effect on bacterial membranes as reported by Oh et al. [65]. The deflection images of sample topography acquired using contact-mode settings allowed for the observation of extensive surface damages of the bacterial outer membrane [65], which confirms the well-established membrane permeabilizing mechanism of this antibiotic [66]. Similarly, Fernandez et al. used atomic force microscopy for imaging the antibacterial effects of tailocins, which are phage tail-like bacteriocins, produced by many bacteria including *Pseudomonas aeruginosa*. The authors noticed the adherence of tailocins to the cell envelope of the phytopathogenic bacterium *Xanthomonas axonopodis*, which resulted in damage and intracellular matrix leakage, leading to the cell lysis. The cell envelope damage was clearly visible with AFM topography. Additionally, AFM was used to obtain phase images and tailocins size, and the acquired size information was similar to the one from TEM [67].

The usefulness of AFM to design and further investigate novel compounds with potent antimicrobial activity against multidrug-resistant bacteria was also encouraged by He et al., who designed a new family of cyclic antimicrobial peptides (CAMPs) targeting MDR strains of *P. aeruginosa*. In detail, AFM and TEM studies showed that CAMP RH11 induces disruption of the bacterial cell membrane, and doughnut-shaped forms of lipid-peptide aggregates were observed around the bacteria. The authors concluded that these shapes suggest the formation of lipid-peptide aggregates at the bacterial membrane or lipid vesicle surface, which leads to bacteria detachment after reaching a critical size, membrane lysis, and finally cell death [68]. Considerable alterations in morphological features as well as cell perturbation of *P. aeruginosa* were also recorded when bacteria was treated with ciprofloxain combined with Lys-a1 antimicrobial peptide [69]. Interestingly, AFM has also been engaged by some researchers to confirm the intracellular mechanisms of actions of newly-synthetized antimicrobials. For instance, Ferreira et al. described the antibacterial activity of a set of fluoroquinolone metalloantibiotics developed by the complexation of fluoroquinolones with divalent metal ions and phenanthroline [70]. The authors confirmed that the designed complex-based antimicrobials exert their activity via bacterial topoisomerase IV and DNA gyrase, and since AFM analyses showed no damage to bacterial membranes, the authors concluded that the probable mechanism of action depends on intracellular pathways only [70]. 

**Figure 3:**
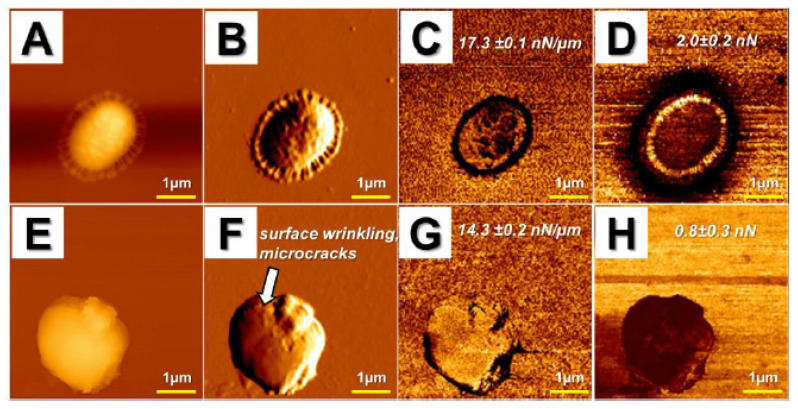
Alterations in morphology and surface properties of CSA-131-treated drug-resistant *Klebsiella pneumoniae* BAA-2473 strain. (**A**–**D**) Control; (**E**–**H**) CSA-131 treated strain; (**A**,**E**) topography images; (**B**,**F**) error signal; (**C**,**G**) stiffness map; (**D**,**H**) adhesion map. Arrow in panel f presents microcracks and surface wrinkling due to CSA-131 treatment.

Likewise, in our research (Figure 3), we used AFM to assess the bactericidal effects of ceragenins (CSA), i.e., synthetic, lipid-based analogs of natural antimicrobial peptides, against NDM-1 carbapenemase-producing *K. pneumoniae* [71]. In a compelling number of studies, ceragenins were demonstrated as potent antimicrobial agents with a membrane-permeabilizing mechanism of action [2,5,72]. In accordance with these reports, we recorded significant activity of ceragenin CSA-131 against the drug-resistant *K. pneumoniae* BAA-2473 strain, which was reflected in membrane disruption, shape shifting, and morphological alterations of bacteria, including surface wrinkling and microcraks formation. Moreover, considerable changes of mechanical properties of the tested bacteria were noted accompanied by a decrease of cellular stiffness and adhesion force. Overall, all of these results obtained on the nano-level extend our knowledge on the utility of ceragenins in the treatment of drug-resistant bacterial pathogens [71]. 

Finally, in one of the newer studies, it was demonstrated that AFM application in microbiological research should also include postmortem analysis of antibiotic-treated bacteria. Such an approach was engaged by Singh et al. In their paper, the authors aimed to examine the bactericidal effect of food-grade lipidic nanoemulsion (noncationized/cationized) using *B. subtilis* as a model bacterium [73]. As expected, TEM, SEM, and AFM analysis revealed nanoemulsion-induced morphological transitions in the treated bacteria, including cell wall destruction and leakage of intracellular content. Nevertheless, only by using AFM was it possible to identify piece-by-piece the fragmented cell wall and to locate it in its appropriate vacant places, thus completing the cell wall contour of the ghost cell. Therefore, AFM studies are particularly important in the context of investigation of the bacterial cell fragmentation mechanisms [73].

#### 2.2.2. Fungi

Considering that the spectrum of drug-resistant strains among fungal pathogens is growing at a dangerous rate and that the number of new antifungal agents introduced into clinical practice is significantly lower than that of new antibiotics, further research on new compounds with potent fungicidal activity is needed. Presently, a growing interest of scientists in the employment of AFM-based techniques for the purpose of novel antifungal development is observed. 

Recently, Quiles et al. used the AFM-FTIR technique to investigate the morphological, mechanical, and biochemical cell wall changes in caspofungin-treated *Candida albicans,* i.e., the leading yeast responsible for fungal infections worldwide. In detail, combining of AFM with infrared spectroscopy revealed mechanisms responsible for caspofungin resistance in *C. albicans* cells, which remodels the cell wall composition and its stiffening through chitin synthesis, proving the usefulness of AFM to understand the drug resistance-associated mechanisms at the molecular level [74]. Another similar study was carried out by Shahina et al. in which inhibitory activity of *Cinnamomum zeylancium* bark extract against *C. albicans* was tested. In this case, atomic force microscopy was used to show the fungal surface exfoliation and loss of the cell wall integrity. In a molecular way, essential oil treatment caused cell cycle arrest by disturbing beta tubulin distribution and triggering cell membrane dysfunction which allowed for the outflow of cellular components [75]. In 2017, Hasim et al. also recorded changes in surface roughness and decreased elasticity of *C. albicans* due to increased exposure of β-(1,3)-glucan, confirming the hypothesis that the therapies enhancing its exposure might be efficiently translated into improved infection control [76]. Similarly, Li et al., owing to AFM, explicitly proved loss of fungal membrane permeability upon treatment with corilagin. The authors witnessed a decrease in the cell’s height and width along with an increase in length and roughness [77]. 

**Figure 4:**
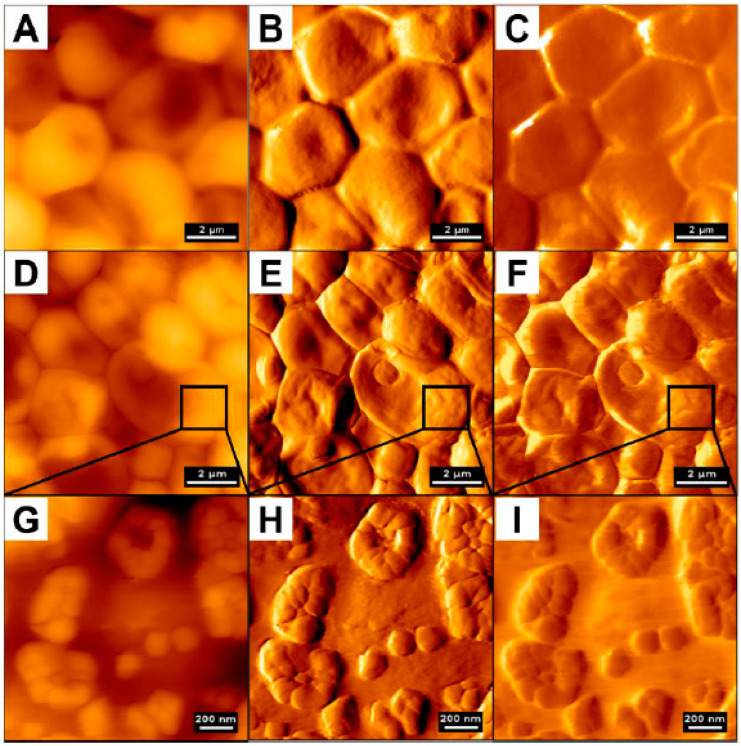
Changes in *Candida albicans* cells morphology upon treatment with cathelicidin LL-37. Panels (**A**–**C**) control; (**D**–**I**) cells treated with LL-37 peptide, 50 µg/mL; (**A**,**D**) topography images; (**B**,**E**) error signal; (**C**,**F**) lateral deflection that corresponds to friction forces (scale bar 2 µm). Panels (**G**–**I**) display local changes in surface morphology of a single cell presented in previous panels (scale bar 200 nm).

In our studies, the atomic force microscope was employed mostly as a tool to investigate candidacidal [3,4] activity of human plasma gelsolin-derived PBP10 peptide [78] and other membrane-active compounds, including ceragenins and human cathelicidin LL-37 (Figure 4) [3,79]. AFM analyses confirmed not only a potent antimicrobial activity of the tested agents, but also provided new insights in membrane-permeabilizing action of ceragenins and LL-37 peptide. In agreement with previous assumptions that AFM should be considered as an important tool to analyze pathogens’ susceptibility to antimicrobial agents, our studies visualized the damage of the membranes of the tested pathogens, which was additionally accompanied by leakage of intracellular content or even whole cell lysis [78,80]. Interestingly, due to the employment of AFM, it was possible to reveal additional aspects of ceragenin CSA-13 and LL-37 killing. Comparison of error signal and lateral deflection images of CSA-13- and LL-37-treated *C. albicans* cells has shown that CSA-13 increases surface wrinkling, while LL-37 causes small clack-like breaks of the cell surface, suggesting that both these compounds might affect the cellular viability via different mechanism. Notably, such information was not available using SEM, which highlights the innovative approach of AFM-based analyses [4].

Some studies also demonstrate the usefulness of AFM-based analyses in examination of the activity of developed antifungals against dermatophytes and other filamentous fungi. In a recent paper, Souza et al., using both AFM and SEM analyses, revealed the potent antifungal activity of a set of synthetic antimicrobial peptides against *Trichophyton mentagrophytes* and *T. rubrum*, which was evidenced by morphological disruption of microconidia morphology, rupture of the cell wall and membrane accompanied by loss of cytoplasmic content, and further cellular death [81]. In a similar way, the capability of AFM to image the filamentous fungi surface upon the introduced antifungal treatment was used by Sen S. and co-workers [82]. 

#### 2.2.3. Viruses

The high resolution of the atomic force microscope has been presented to allow the visualization not only of a micrometer scale pathogens, i.e., bacteria and fungi, but also to permit the investigatation of pathogens with nanometer sizes, such as viruses. 

**Figure 5:**
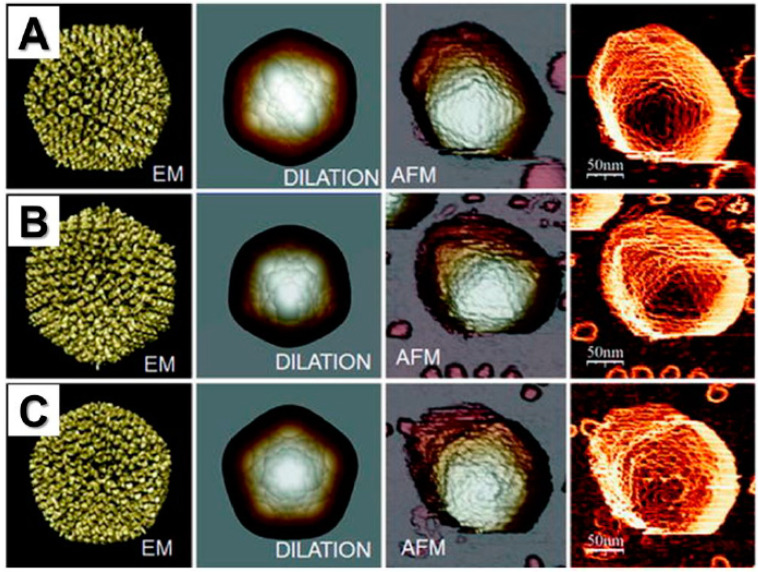
Images of human adenovirus collected using AFM show the topographies corresponding to single adenovirus particles oriented with a (**A**) twofold, (**B**) threefold, or (**C**) fivefold symmetry axis on top. AFM images are compared with EM and EM-dilated structural models. The right column (fourth column) shows AFM topographic images that have been filtered to enhance the borders by obtaining the cosine of the angle between the normal vector of the surface and the normal direction of the paper sheet. Adapted with permission from Springer [77].

For example, Godon et al. described how to record nonbiased topographical surfaces of viruses [83]. The authors tested tobacco mosaic virus in various conditions such as: Air, liquid, imaging on mica or self-assembled monolayer, and two different imaging modes: Tapping mode and PeakForce tapping. The key factor in air measurement was the substrate, whereas in liquid it was the imaging mode. Godon et al. acquired the anticipated height of the virus with PeakForce tapping in both environments, but only when using the self-assembled monolayer. This study suggests that the best imaging can be acheived by switching from mica to self-assembled monolayer. Another research group led by Kämmer presented the advantages of using AFM instead of SEM [84]. In the referred study, the authors used *Herpes simplex* virus to identify differences between the two methods. The results show that the SEM, but not AFM, measurement lowered the dimensions due to the samples’ preparation process. As a result, AFM could play a key role as a forefront technique in diagnostic virology. Similar comparison of SEM and AFM was performed by de Pablo et al. [85]. The example image of virus topography compared to electron microscopy is shown in Figure 5.

Furthermore, Barinov et al. presented the use of high-resolution AFM imaging to extract and create height and volume distribution histograms to describe the oligomeric state of hemagglutinins of the influenza virus, which are glycoproteins causing agglutination of red blood cells [86]. The results showed that the large oligomers were unstable, and the oligomeric size was affected by pH and ligands. The DNA aptamer induces the formation of large oligomers, whilst antibody binding results in generation of small oligomers [86]. Another study by Azinas et al. showed that membrane-containing virus particles behave similarly to composite materials [87]. AFM was used for topography and nanoindentation assays to assess their stiffness and yield behavior against mechanical stress, which is higher in viruses that lack a membrane outside their capsids. Another study performed on viruses presented the real time self-recovery of the membrane. As demonstrated, the authors damaged the protein shells with single nanoindentations or by increased interaction force between the cantilever and a shell in the amplitude modulation dynamic mode, and then recorded the self-recuperation events for T7 bacteriophage capsids. Notably, regardless of the considerable limitations of the research, including low statistical significance, low number of particles tested, and no control over what type of damage would be restored, this study was a first to demonstrate the utlity of AFM in investigating fracture self-healing on virus shells [88]. 

### 2.3. Alterations in Microbes’ Cellular Stiffness as an Indicator of Antimicrobial Activity of Tested Molecules

The measurements of microbial cell physicochemical properties help evaluate their interactions with the environment and with each other. It has been proposed that properties of live microbial cells are modifiable by antimicrobials and antiseptics agents [3,4,89]. Viscoelasticity, and adherence of cell to surface, and cell to cell, have been recognized as factors promoting microbial survival [90,91]. New nanoscale evaluation of microbial properties is providing a new path for research into growth and survival of microbes, as well as they eradication upon action of antimicrobial agents [92,93]. 

**Figure 6:**
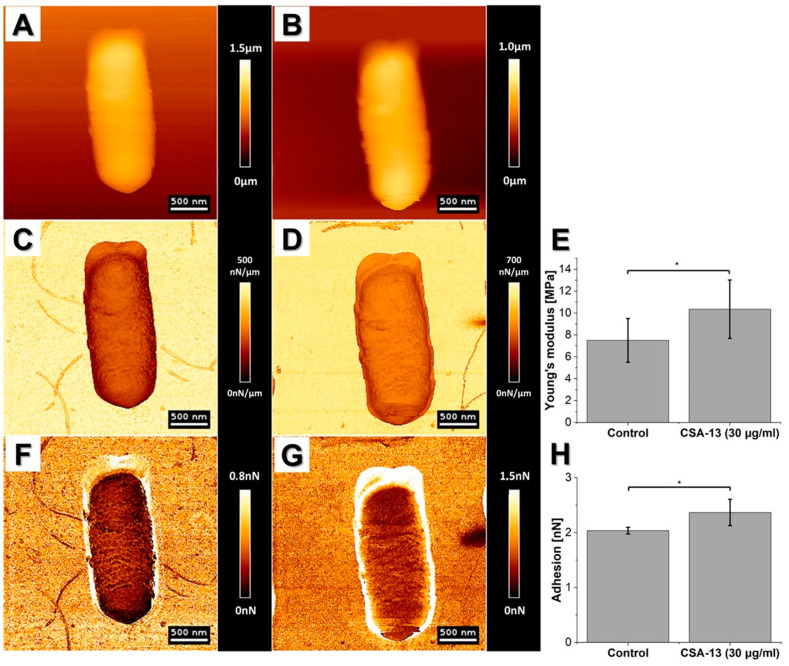
Changes in mechanical properties of *Bacillus subtilis* cells before and after CSA-13 (30 µg/mL) treatment. (**A**,**C**,**F**) Control and (**B**,**D**,**G**) after CSA-13 treatment. (**A**,**B**) Changes in height of the cell; (**C**,**D**) stiffness map; (**E**) average Young’s modulus; (**F**,**G**) adhesion map; (**H**) average measured adhesion force. Unpaired Student’s *t*-test was used to confirm statistical differences between the samples (* *p* ≤ 0.05). More detailed information of AFM application in the study aiming to understand the rheological consequences of antibacterial agent binding/insertion into *B. subtilis* cell membrane are presented in [17].

In 2003, da Silva et al. tested the effects of peptidyl-glycylleucine-carboxyamide (PGLa), an antimicrobial peptide isolated from hemocytes of frog skin, on *E. coli* viability. According to the provided AFM-based analyses, which included imaging and measuring bacterial stiffness in physiological conditions upon treatment with this agent, da Silva demonstrated that the PGLa activity is manifested by changes that occurred over two phases. The first one was characterized by the loss of outer membrane stiffness and subsequently of topographic features, as well as the formation of micelles. The second phase consisted of further cell damage and loss of cytoplasm material, followed by cell detachment from the substrate and lysis of treated cells which indicated the likely PGLa interaction with membrane and its cross-binding to the negative charges of lipopolysaccharide (LPS) [94]. The “two-phase effect” was also observed in one of our studies aimed to decipher the antimicrobial action of cathelicidin LL-37 or ceragenin CSA-13 against *Bacillus subtilis* [17]. Briefly, initally we recorded stiffening of *B. subtilis* cell envelope upon treatment with these agents, which afterwards softened in a time-dependent manner in comparison to non-treated cells [17]. The former phenomen is most likely an active bacterial response against the antimicrobial assult, which is in line with observations that bacteria have the ability to regulate the stiffness according to their needs, and might indicate the importance of cell stiffness regulation as a part of bacteria cell survival. Overall, these obervations contribute to a better understanding of the rheological consequences of antibacterial agent binding/insertion into the bacterial cell membrane (Figure 6). 

In other research, considerable changes in the antibacterial effect of SiO_2_-NPs nanoparticles depending on their size were noted [95,96]. These studies showed that 100-nm SiO_2_-NPs lack the ability to modify both morphology and *E. coli* cell stiffness to a significant level, indicating their harmless behavior towards those bacteria. However, 4-nm nanoparticles presented with a significant decrease in the Young modulus, which is assumed to be associated with damaging the bacterial outer membrane and the destruction of the peptidoglycan layer which subsequently leads to the cell lysis [95].

Most recently, AFM was also employed to assess the morphological and mechanical properties governing bacteria antibiotic resistance and persistence [97]. Using *E. coli* as a model bacterium, Uzoechi et al. performed AFM-based analysis of morphology, adhesion, elasticity, root mean square roughness, and surface thickness of ampicillin-treated bacteria and concluded that both resistant and persistent *E. coli* bacteria combat the ampicillin exposure by decreasing the cellular size, introducing into the dormancy state, and altering the mechanistic features of cells by increasing their elasticity, roughness, and grafting density. Notably, knowledge about such mechanistic insights into resistant and persisting bacteria functioning in response to antibiotic assault is crucial for further development of new antibiotics [97]. Interestingly, in one of the recent studies, Krce et al. demonstrated that antimicrobial-treated bacteria might exert differences in mechanical properties, even when inspection of cellular morphology does not detect any alterations, as concluded from their research aimed to probe *E. coli* bacteria treated with silver nanoparticles [98]. Since the comparison of morphological features of untreated and treated bacteria did not provide any significant data, authors aimed to examine mechanical properties of the cells using the QI mode. Interestingly, it was recorded that Young’s modulus was distributed binomally, with two clearly split maxima that differed in value for an order of magnitude. Particularly, the softer regions were randomly distributed on the cell, which speculatively might represent the future points of pore formation. For post-treated bacteria, the narrowing of the stiffer Young’ modulus distribution was also recorded, which was explained as an indicator of metabolic activity reduction [98]. 

The effects of another antimicrobial peptide—Psd1 defensin—were tested on *C. albicans*, demonstrating surface alterations, membrane disruption, and leakage of cellular contents accompanied by cell softening upon Psd1 treatment [99]. Notably, changes in cell stiffness were the first indicator of the defensin’s effect and can be related with the evidence that Psd1 has glucosylceramide as a molecular target in *C. albicans* cell membrane [100]. 

**Figure 7:**
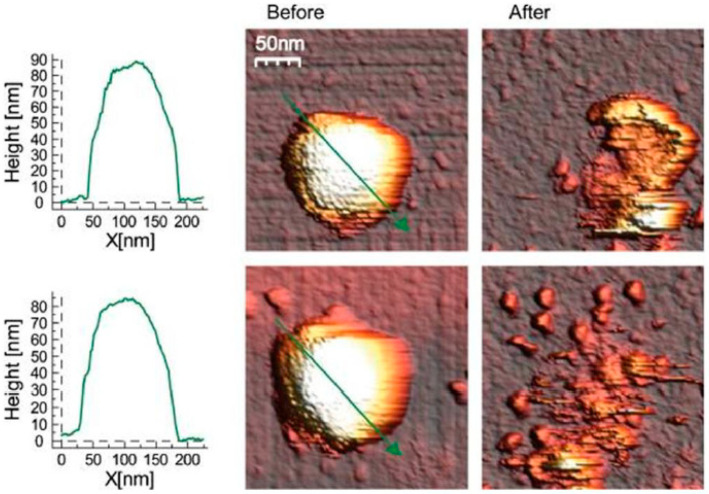
Structure of human adenovirus type 5 (AdV) capsids before and after indentation with a cantilever. The images on the right contain the same capsids but after indentation, representing the damage inflicted by the cantilever indentation. Reprinted with permission from Springer [101].

In terms of AFM employment to study viruses, van Rosemalen et al. determined the alterations in the mechanical changes of human adenovirus type 5 (AdV) upon the induction of single point mutation [101]. It was demonstrated that even single-point mutation might result in the two-fold increase in stiffness, which authors suggest is due to the DNA crosslinking activity of protein VII, and which may help to develop more stable vectors for therapeutic applications. The structure of AdV capsids before and after measurement is presented in Figure 7. 

### 2.4. AFM Analysis in Investigation of Microbial Surface Adhesion, Colonization Mechanisms, and Virus–Cell Binding

Adherence governs the ability of bacteria to colonize different surfaces. For this reason, the limitation of adhesion process is favorable and highly encouraged in the course of the creation of novel biomaterials, both for external use and implanted, as well as the development of anti-infectious therapeutics or production of improved materials for industrial purposes [30,44,102]. The capability of AFM to measure adhesion forces between the catilever tip and scanned sample is particularly useful in this manner. 

Measurements of adhesion have to take into consideration the influence of capillary condensation of water [103], because capillary forces between the AFM tip and the wet surface could interfere with imaging and force measurement. These forces could be avoided by operating the whole experiment immersed in solution, which also allows for in situ imaging [104]. Measurements in the air help avoid the possibility of suspended particles’ and bacterial cells’ attachment to the tip. On the other hand, physiochemical changes to the cells may occur during the drying process [105]. 

#### 2.4.1. AFM Analysis of Mechanisms of Pathogens Colonization, Microbe–Microbe Interactions, and Binding to Cells

Researchers can use single-cell force spectroscopy (SCFS) and single-molecule force spectroscopy (SMFS) as tools for adhesion force measurement. Compared to isothermal titration calorimetry (ITC) and surface plasmon resonance (SPR), force nanoscopy enables the label-free analysis directly on live cells [106]. Importantly, the adhesion measurements allow us to better understand the microbial adhesion and colonization processes, which might be further translated into improved materials for medicine or industry. The review article that describes those methods in detail was recently published by Beussart et al. [107].

In one of such studies, Sjollema et al. used AFM to investigate the attachment and re-attachment of various bacteria from *Staphylococcus* and *Streptococcus* species to tested surfaces using the SCFS mode with a PLL-coated bacterial probe. Results demonstrated that bacteria adhere to surfaces through multiple tethers that can detach and re-attach, but never at the same time, which leads to irreversible bacterial adhesion [45]. In another study, Wang et al. studied the growth and adherence of *S. aureus* in the presence of prostaglandin E2 (PGE_2_), i.e., a key inflammatory mediator in chronic infections [108]. Authors used PLL-coated tipless cantilevers to attach bacteria (untreated and treated with PGE_2_), measured surfaces with and without human fibronectin, and showed higher adhesion forces to human fibronectin after treatment with PGE_2_, which confirmed that *S. aureus’* growth and adhesion to epithelial cells was promoted by the COX-2/PGE2 pathway [108]. Most recently, AFM-based single-molecule experiments also allowed for the investigation of the mechanical strength of *S. aureus* protein clumping factor A (ClfA) binding to endothelial cell integrins, i.e., proteins playing a crucial role during sepsis. As demonstrated, adhesion forces between single bacteria and tested integrins are strongly inhibited by an anti-αVβ3 antibody, which not only provided additional data on *S. aureus* virulence, but also has important implications for the design of new therapeutics against this pathogen [109].

Recently developed fluidic force microscopy (FluidFM) technology can increase the efficiency of AFM assays in examination of bacteria adhesion [110]. FluidFM opens the possibility to either extract cellular material or deliver exogenous substances into the cells. Moreover, FluidFM can be used for single-cell immobilization by creating under pressure transportation, and can be released at a desired location. Cell fixation by suction allows us to perform whole-cell adhesion measurements and a combination of both cell fixation and solution insertion by microchannel direct changes to adhesive properties in whole-cell adhesion measurements. Beaussart et al. established the AFM-based nanoscopy method to assess antiadhesion activity of multivalent mannofullerenes directed against *E. coli* FimH [30]. In this experiment, the thiol bond was used as a linker between the anti-adhesion compound and AFM tip. Noticeable was the presence of multiple ruptures and plateau events, demonstrating that the separation of bacteria from the mannose surface leads to the unfolding of pili. This emphasizes the influence of the experimental set-up on the mechanical response of piliated bacteria. Additionally, the authors suggested that stretching pili multiple times through FimH-mannose bonds leads to their denaturation, which decreases the overall adhesion of the cell surface. All of the above revealed the strong anti-adhesion effects of *E*. *coli* to the carbohydrate receptors by glycofullerenes, which is an encouraging tactic for anti-adhesion-based therapies [30]. Moreover, AFM might be employed as a platform for quantifying the activity of anti-adhesion compounds directly within bacterial suspension. Additionally, employment of Beaussart’s nanoscopy method might help with designs of new anti-adhesion drugs [30,110]. Furthermore, increasing the throughput of single-cell AFM assays with FluidFM could yield statistically relevant data within a few hours.

**Figure 8:**
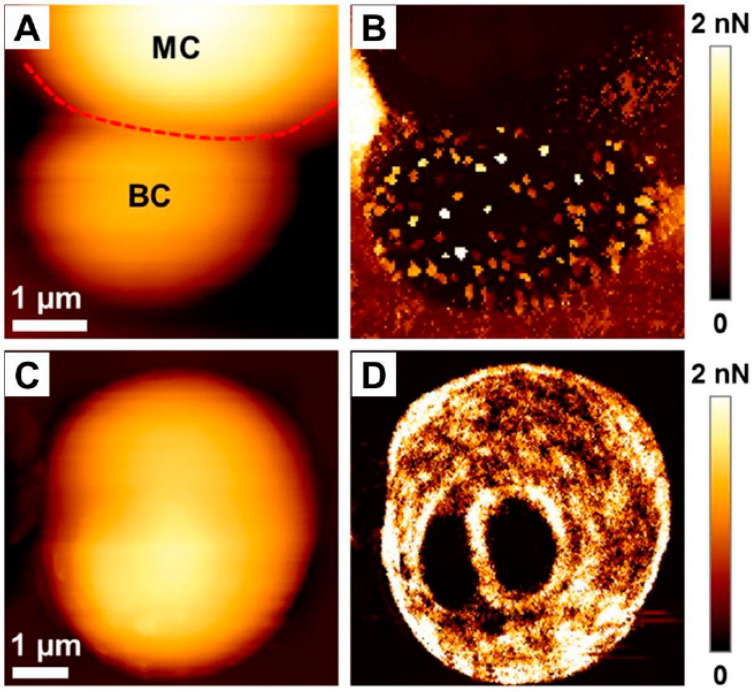
Adhesive properties of *C. albicans* cells. (**A**) Height image of a budding *C. albicans* cell in a polydimethylsiloxane stamp, and (**B**) adhesion image corresponding to the height image. In (**A**), MC stands for mother cell, BC stands for budding cell, and the red dotted line represents the demarcation between the two different cells. (**C**) Height image of a single *C. albicans* cell exhibiting two bud scars, and (**D**) adhesion map corresponding to the height image. Adapted with permission from Elsevier [111].

High resolution of AFM and its capability to analyze the force signature of single proteins on single cells also allows for a better understanding of the adherence and virulence factors of *C. albicans*, as demonstrated by Formosa et al. [111]. *Candida* species are considered as opportunistic pathogens; thus, in order to colonize and propagate in the blood stream they often adhere to various substrates, mostly those used to build medical devices [111,112]. In a study by Formosa et al., data were recorded in the quantitative imaging mode (Figure 8), demonstrating that the adhesins at the cell surface were organized in nanodomains composed of free or aggregated mannoproteins. Moreover, the authors mention that the cell wall was permanently remodeled as a reaction to the environment, which made the reproduction of experimental conditions challenging [111]. In addition to that, combining atomic force microscopy with genetic tools also allowed us to understand the mechanisms governing the adherence of fungal cells to the abiotic surface in order to initiate biofilm growth. Using such an approach, Valotteau et al. concluded that EPA proteins, being a family of lectins mediating the adherence of *C. glabrata* fungi to host glycans, were simultaneously responsible for nonspecific hydrophobic and hydrophilic interactions with abiotic surfaces. In this aspect, AFM was used to quantify the forces between single *Candida* cells and hydrophobic/hydrophilic substrates. As demonstrated, silencing of *EPA* genes had a dramatic effect on surface adhesion, which confirmed the hypothesis of researchers [113]. 

AFM-based adhesion measurements were also reported to be useful in investigation of microbe–microbe interactions, which helps to explain the protective effects of some microorganisms against pathogenic-induced diseases. In this persepective, most recently Meng et al. examined adhesion-based binding of *P. aeruginosa*, being a common foodborne and waterborne pathogenic bacterium, to *Geotrichum candidum* LG-8, i.e., a fungus isolated from a kefir and used as a probiotic component [114]. Although in relation to the morphology of bacteria-treated fungus AFM did not provided any additional data when combining with TEM and SEM, and as such is recognized only as a complementary technology for surface characterization of fungi associated with bacterial adhesion, its capability to record alterations in surface roughness was crucial to more deeply assess the mechanism of bacteria–yeast binding. Notably, the authors detected that bacteria-induced nanoscale changes in the roughness of the fungal LG-8 surface played a role in assisting the adhesion process [114]. Another study was carried out by Ma et al. and showed the effects of bacteriocins released by *Streptococcus sanguinis* on the mechanical properties of *C. albicans* [115]. *S. sanguinis* is a well-known dominant bacteria in healthy human oral cavity, reported to help to exert an antagonistic effect on *C. albicans,* thus protecting the oral cavity against fungal-induced disbalance of microbiota. To date, only some considerable changes in morphology of bacteriocin-treated *Candida* cells have been reported [116]. AFM analyses revealed that Young’s modulus of *C. albicans* was reduced by the presence of *S. sanguinis* bacteriocin, resulting in an increase of elasticity and deformation ability. Analogical observation was noted for adhesion ability, which deceased after treatment with bacteriocins. All of the measurements were performed with tapping mode [115].

Although the number of research papers focusing on the determination of interactions of viruses with host cells using AFM is considerably lower than in the case of bacteria or fungi, a few interesting papers have recently been published. In the first one, Newton et al. established a method to assess the virus binding to cell surfaces using confocal microscopy combined with AFM, allowing for subsequent quantification of binding events and observation of them. Overall, this novel approach depends on probing specific interactions with cells expressing viral cognate receptors with further measurement of the affinity of this interaction, and may provide some new data on early stages of cell–virus interactions [117]. In another study, Lin et al. studied the unbinding events of HPV16/anti-HPV16 pairs with AFM [118]. The authors used a functionalized tip to observe the binding/unbinding events and measure the forces between HPV16 tied to AFM cantilever and anti-HPV16 coating on the surface. Remarkably, due to this approach the authors could define if the patient had HPV just from the results of unbinding forces and the distribution of stiffness. In addition, this method was suggested as beneficial in studies of the possible role among subtypes of HPV in oncogenesis of cervical cancer [118]. Importantly, AFM-based analyses were demonstrated to be highly valuable in the investigation of mechanisms of infection of cells by severe acute respiratory syndrome coronavirus 2 (SARS-CoV-2). Yang et al. investigated the binding events of SARS-CoV-2 with angiotensin-converting enzyme 2 (ACE2) receptor, i.e., one of the critical receptors for virus entry into host cells. The authors, through force-distance curve-based AFM, demonstrated the kinetics and thermodynamics of interactions between ACE2 and S glycoprotein of SARS-CoV-2 virus, expanding the knowledge about coronavirus pathogenecitiy and suggesting a strong therapeutic target for COVID-19 treatment [119]. 

#### 2.4.2. Utility of AFM in Fabrication of Biomaterials with Anti-Adhesive Properties

Adhesion measurements using AFM are also particularly useful during the production of improved types of materials, for which contamination with microbes with further formation of biofilms of their surface would be unfavorable or patient endangering. In this regard, Aguayo et al. conducted a study to assess the early adhesion of *C. albicans* to dental acrylic surfaces [120]. In another study, Ozel et al., due to AFM evaluation of *C. albicans* and *S. mutans’* adherence to provisional crown materials, detected differences in the surface roughness and chose the appropriate material for clinical introduction. During the course of the study, the authors observed that microbial colonization is initiated in grooves, gaps, and recesses on the surface. AFM imaging revealed that more peaks and groves were spotted in groups with polymethyl methacrylate (PMMA) content, while shallow pits and bulges appeared in bis-acrylic groups, resulting in the lowest (PMMA) and highest (bis-acrylic group) adhesion [121]. At the same time, Vargas–Blanco et al. used AFM-based methods to focus on coatings for medical devices that could prevent the attachment of *C. albicans* [122]. In this study, filastatin, which inhibits adhesion of *C. albicans*, was applied on different biomaterials such as bioactive glass, silicone, or dental resin. Adhesion to these biomaterials was measured by direct visualization of fluorescence the day after wild-type *C. albicans* staining and AFM [122]. 

In terms of implanted biomaterials, the limitation of pathogenic colonization, and, thus, the decrease of biofilm-derived bacterial and fungal infections, is strongly advantageous. To address this issue, Alam et al. tested forces between *S. aureus* bacteria immobilized on the tipless cantilever and biomaterials such as titanium alloys (Ti-6AL-4V), hydroxyapatite (HA), stainless steel (SS), and ultra-high molecular weight poly ethylene (UHMWPE), i.e., materials commonly used in the production of bone implants [44]. The tipless cantilever was coated with PLL for 2 min, then the coated cantilever was immersed in bacterial suspension, and fluorescence microscopy was performed to confirm that the single live bacteria was attached to the cantilever. Image analysis indicated that surface energy, roughness, and wettability played a vital role in bacteria adhesion. Further investigation presented UHMWPE and HA to be the best choices in terms of the lowest bacterial adhesion forces. Additionally, HA was presented as a better bioactive bone replacement material due to its antibacterial properties [44]. Recently, Carniello et al. used the SCFS technique and bacterial-immobilized AFM probes as an indicator of mechanical stress to investigate how chemical stresses combined with high adhesion forces between *S. aureus* and biomaterial surface influence drug resistance-associated gene expression. The authors concluded that stronger adhesion forces accompanied by the presence of chemical stressors resulted in upregulation of expression of drug resistance-determining genes. Thus, the development of biomaterials with anti-adhesive properties would not only serve to decrease biofilm-derived infections occurrence, but would also limit the spread of microbial drug resistance [123,124]. Most recently, AFM-based analyses of surface roughness of coating layers established the utility of electrochemical polymerization-induced poly(3,4-ethylenedioxythiophene) derivative nanohybrid coatings on stainless steel to obtain anti-fouling and anti-biofilm biomaterial for production of cardiovascular stents and surgical apparatus [125]. 

#### 2.4.3. AFM Analysis of Material Surfaces for Industrial Purposes

The utility of atomic force microscope for industrial purposes was also established, particularly due to its capability to characterize materials’ surface properties such as surface roughness (R_a_). In one study, AFM measurements allowed the authors to compare the changes in water quality and formation of biofilms in copper, garlanized steel, and plastic pipes used for water distribution systems. Accordingly, it was concluded that copper and galvanized steel had the highest roughness, whilst the observed adhesion was highest for galvanized steel but no cell counts were obtained from copper samples, which might be due to bactericidal effect of copper ions [126]. However, studies have shown that after a long period of time there is no difference between copper and plastic materials [127,128]. In another study, Assaidi et al. tested the adhesion changes of *Legionella pneumophila* serogroup 1 and serogroup 2 to different materials used in water systems [102].

#### 2.4.4. The Employment of Atomic Force Microscopy in the Investigation of Microbial Biofilms

Infections associated with biofilm formation on the surface of implants, medical devices, catheters or food plates represent a major problem in surgical procedures, infection treatment, and recuperation. They constitute a serious risk for patients in the hospital environment [129,130]. Thus, limiting the survival of biofilm-embedded pathogens grants an opening to reduce the number of microbial infections [131]. To date, AFM-based measurements of adhesion [129], surface roughness [130,132,133], topography [133,134,135,136], nanomechanics [135], and the stiffness of biofilm [137] were succesfully useful in regard to microbial biofilm investigation. Nevertheless, an ever-growing number of studies has confirmed that those applications should be extended by examination of biofilm structures and assessment of biofilm susceptibility to antibiotics.

In one of the studies, Nielsen et al. confirmed using AFM that due to antibacterial features of isoeugenol, a simple coating of a surface with this essential oil can thwart biofilm formation on stainless steel and polyethylene surfaces, which may result in reducing the spreading of microbes through hotspots such as tables, sinks, toilet seats in hospitals, or in homes on chopping boards, and even with possible applications on medical implants [129]. For this hypothesis testing, the authors used a tipless cantilever coated with isoeugenol and recorded the retraction forces from bacteria placed in a petri dish. Additionally, AFM images of uncoated and coated stainless steel were acquired to observe height and adhesion force [129]. 

While Nielsen et al. focused on hotspots in the hospital and home environment, Gonçalves et al. developed antibacterial coatings specifically designed for medical purproses [130]. To that end, phosphotungstate organically modified silicate (ormosil) was dopped with core-shell nanoparticles (SiO_2_@TiO_2_) and combined in the coating with silver nanoparticles included by photoassisted synthesis. Surface morphology and roughness R_q_ measurements before and after irradiation of phosphotungstate ormosils with silver cation were acquired using AFM working in the tapping mode. The results showed that ormosils with silver nanoparticles could achieve eradication of *P. aeruginosa* and *S. aureus*, in contrast to unmodified gold nanoparticles. Moreover, ormosil-modified nanosystems were noted to easily adhere to indwelling materials without any harmful effects on vascular cells. Notably, antimicrobial effects of developed nanoformulation remained at least three reutilization cycles in very aggressive condition, which makes them a promising strategy to develop self-sterilizing materials [130]. 

Another study by Quatrin et al. assessed the antimicrobial and antibiofilm activities of nanoemulsions containing *Eucalyptus globulus* oil against three species of *Candida* using AFM topography [134]. Inorganic nanoparticle/teflon-like (CF_x_) composites were tested as antimicrobial surfaces by Sprotelli et al. using AFM in the air dynamic mode [132]. AFM studies on Ag-CF_x_ composite film with incubated *Pseudomonas fluorescens* compared to the control sample showed increased surface roughness on the bacterial outer membrane and cells with lower density, which indicated the anti-bacterial efficiency [132]. Since oral biofilms also represent a challenge in the treatment of infections, de Souza et al. subjected a dental biofilm to *Melaleuca alternifolia* (TTO) and nanoparticles of TTO (NPTTO) and confirmed considerable alterations in the membrane structure of biofilm-embedded cells using AFM [138]. 

AFM was also used to detect biofilms formed by anaerobes and atypical mycobacteria isolated from hospital patients [139]. Recently, nanoresolution of the atomic force microscope and AFM-based morphological analysis was also reported to be useful in investigation of *K. pneumoanie* biofilms, notably by combining AFM with infrared (IR) spectroscopy and spectral imaging, which, overall, allowed for the collection of data on the composition and distribution of the chemical components of biofilm [140].

While the problem of the growth of fungal biofilms on the surface of materials for both medical and industrial purposes has been neglected for many years, it can no longer be denied that their formation causes significant economic losses to the food and health sectors. The issue of food contamination by fungal biofilms drew the attention of Handorf and colleagues, who aimed to evaluate the anti-biofilm effects of a microwave-induced plasma torch treated against *Candida* biofilms [141]. Since conventional methods of biofilm removal, including brushing or mechanical removal of the biofilm with high-pressure washers, as well as biocide-based treatments, are insufficient, it is extremely important to combat microorganisms embedded in biofilm with newer methods. In this respect, AFM is a very valuable tool for studying the morphology and structure of a biofilm [141]. 

## 3. Investigation of Bacteria and Fungi Phenotypic and Virulence Features Using AFM

The wide application possibilities of the atomic force microscope, including the ability to visualize samples and to perform measurements of mechanical properties in the same area of bacteria, opened new analytical possibilities in the field of microbiological tests and enabled the assessment of phenotypic features of bacteria and the study of virulence factors. More recently, Marshal et al., using AFM-based morphological and nanomechanical analysis, identified the surface properties of bacterial polysaccharide capsules that are required to avoid host-mediated immunity and determine the virulence of many microbes, including *S. pneumoniae* and *S. mitis*, i.e., pathogens inhabiting the human respiratory tract. Force-volume mapping using AFM combined with biochemical analyses demonstrated that identical capsular serotypes of bacteria present similar biomechanical characteristics, independent of bacterial strains, and that might be further translated into data on virulence phenotypes [142]. Mechanical forces are also crucial for proper peptidoglycan synthases and hydrolytic enzymes activity and, thus, for mycobacterial cell division as demonstrated by AFM imaging, nanomechanical mapping, and nanomanipulation combined. This confirms that studying the molecular mechanisms of bacteria physiology should be performed with subsequent investigation of physical factors affecting the cells [143]. Considerable morphological and mechanical alterations were also noted for Burkholderia cenocepacia strains isolated from the patients in different stages of cystic fibrosis (CF) [144]. To date, it has been established that pathogens associated with long-term lung infections in CF patients face a spectrum of stressful environmental factors, particularly due to action of the immune system, antimicrobial therapy employed, or a decrease of oxygen availability, resulting in phenotypic diversification of pathogens in terms of antibiotic resistance, biofilm-forming abilities, and virulence potential [145]. Hassan et al. demonstrated that along with CF progression, the cell height and shape of *B. cenocepacia* changes from rods to cocci, which is favorable for the reduction of the cell surface sensitivity to immune cells due to the smaller ratio surface/volume. This process is additionally accompanied by a decrease of elasticity modulus, which suggests the essential role of cell wall nanomechanical features in this adaptation process [144]. Regarding the example of *Serratia marcescens* CH-1 cells, Lin et al. also demonstrated that AFM might be a valuable tool to define the structural arrangement of transmembrane structures on integral prokaryotic bacteria, rather than on an isolated membrane. Notably, the authors characterized types of membrane pores, providing a know-how in the investigation of three-dimensional membrane pore structures and their functions on living prokaryotic cells, which is currently inaccessible by conventional microscopic observations [146]. In another study, Liu et al. used single-molecule force spectroscopy to investigate the interaction between *S. aureus* bacteria and the cell wall-binding domain (CBD) of bacteriophage lysins, i.e., specific peptidoglycan hydrolases mediating the lysis of host bacterium. The performed SMFS analysis not only provided new data on the binding properties of lysin CBD with bacterium, but also highlighted the possibility to use it in bacterium detection, as well as a therapeutic target for anti-CBD antibodies. Particularly, the possibility to develop specific bacteria-targeting antibodies would be advantageous, since antibodies currently used for *S. aureus* detection are characterized by a high cost of production and poor stability [147]. Recently, Iqbal et al. engaged AFM nanoscale and phenotypic analysis of *Yersinia pestis* bacteria cultured within soil matrices, being the most important reservoirs for its spread. Coupling AFM with biochemical profiles of bulk populations using fatty acid methyl ester profiling (FAME) allowed for a better understanding the persistence of this pathogen within environmental matrices [148]. Due to AFM analysis, it was possible to better characterize the morphological alterations of Salmonella bacteria entering the viable but non-culturable state (VBNC) upon exposition to stress conditions routinely present in food environments, i.e., low temperatures and high concentrations of sodium chloride [149]. As demonstrated, VBNC bacteria reduced their size and changed the morphology from bacillary to coccoid. At the same time, no significant alterations were observed in the presence of acid and oxidant compounds, which provide new data for the purpose of further improvement of food safety [149].

Regarding the investigation of bacterial spore structure and functioning, a particularly interesting study was performed by Liu and co-workers [150]. The authors engaged AFM-based SMFS to investigate the specific interactions between *B. subtilis* spore’s coat proteins, determining the spores’ resistance to unfavorable environmental conditions and analyzing both unbinding force and kinetic data in CotE and CotZ proteins. As demonstrated, the morphogenetic protein CotE interacted directly and specifically with CotZ, resulting in the formation of a stable complex, and this phenomenon was strongly dependent on the CotE/CotZ ratio. Importantly, the above results not only provided crucial information on spore cote assembly, which might be translated into the design of improved sporicidal agents, but also confirmed unique advantages of the AFM/SMFS analysis for characterization of other coat proteins [150]. 

## 4. Physicochemical Characterization of Developed Antimicrobials Using AFM

The possibility of employment of AFM-based measurements for detailed characterization of material properties in nanoscale allows for the physicochemical evaluation of synthetized compounds. In this regard, AFM is mostly employed during nanoparticles’ synthesis and development of nanosystems with a broad spectrum of antimicrobial activity. 

Nanoparticles (NPs) and nanosheets have been of interest for many years now, due to the possibilities in biomedical and industrial technologies [151,152]. The short- and long-term effects of nanoparticles are still a vastly researched topic [153,154,155,156]. The most utilized nanoparticles with antimicrobial properties are silver nanoparticles, particularly due to the potent bactericidal activity of silver itself and their relatively low toxicity [157,158,159]. Nevertheless, a broad spectrum of other metal-based nanoparticles, particularly made of cerium (IV) oxide, copper, titanium dioxide, cadmium sulphide, zinc oxide, and gold, is gaining considerable interest from scientists [78,160,161,162,163,164,165]. AFM can be used as an important tool when it comes to analyzing NPs, and topography, height, sorption, structure, dispersion, and agglomeration can be acquired with AFM [159,162,166,167]. Data can be acquired with the classical force volume mode or the proprietary quantitative imaging (QI™) mode of JPK Instruments™, the Peak Force™ tapping mode of Bruker™, or “jumping mode” of Nanotec™. Additionally, it is a good tool to confirm any bacterial or fungal cell envelope changes occurring upon treatement with tested nanoparticles [4].

The number of reports presenting the possibility of using AFM in the physicochemical analysis of the obtained nanoparticles is overwhelming; therefore, only some of the papers that have been published recently will be cited. One interesting study was made by Lu et al. who approached it with the idea of changing the alignment of graphene oxide to enhance the antibacterial activity, and the change of the alignment was confirmed with AFM [168]. Another author also used GrO, but with directly functionalized tryptamine (TA). The functionalization was confirmed in many ways including AFM with the use of tapping mode to acquire topographical imaging. The results show that the addition of TA provided stable antimicrobial coating characterized by low toxicity and high biocompatibility [169]. Shaheen et al. proposed the large-scale production of silver nanorods via cellulose nanocrystals. The surface topology, particle size, and overall size distribution was performed with AFM [170]. Dobrucka et al. also employed AFM for ZnO nanoparticles’ topography, and then tested them against bacteria and yeast [171]. 

## 5. AFM as Novel Tool to Improve Currently Used Diagnostic Methods

Current research into new and improved ways of diagnostics could be enhanced by the use of AFM [172,173,174]. Apart from its usefulness as a nanoresolution tool for the evaluation of morphological changes [4], stiffness alterations [99], and adhesion forces, AFM can also be successfully used for the detection of bacteria, even at microbial smallest concentrations [175]. Kasas et al. used a microfabricated 200 µm-long AFM cantilever (DNP-10 Bruker) to observe the oscillations of the lever while bacteria or yeast were attached to it. The lever was functionalized with glutaraldehyde to achieve the best immobilization efficiency. The authors observed that the living *E. coli* induced a large fluctuation of the sensor, and that this was reduced upon 15 min of treatment with ampicillin, as well as with ciprofloxacin and caspofungin when *S. aureus* and *C. albicans*-containing samples were tested, thus indicating microorganisms’ susceptibility to antibiotic addition. In effect, these observations indicate a great potential of AFM as a tool for quick MIC/MBC assessment in comparison to standard clinical microbiology methods [176].

In a similar study carried out by Etayash et al. [175], differences in fluctuations between live and dead bacteria were detected after attaching bacteria to the lever with the use of the microchannel that was coated with antimicrobial peptide Leucocin A, in order to selectively interact with bacteria-targeted receptors. In such a design setting, bacteria were forced to pass through the microfluidic channel, and then the bacterial adsorption to the surface resulted in changes in the resonance frequency and cantilever deflection. Furthermore, the excitation of attached bacteria with infrared radiation changed the cantilever deflection proportionally to infrared absorption by the bacteria inside the channel, thus creating a nanomechanical infrared spectrum for selective identification. Notably, the resonance frequency changed depending on the live or dead state of the bacteria, which could be used in testing of antimicrobial effects. As the authors noted, the measurement could be enhanced by confocal microscopy to assess cell viability using staining, and, more importantly, the testing might be performed even at concentrations of a single cell per µL [175].

## 6. Summary

Imaging at a high resolution is of great importance in biology, since basic life processes occur at the nanoscales. Due to its advantages such as working in liquid with high resolution on living cells, the AFM measurements cannot go unnoticed or seen only as a powerful imaging tool, since AFM is also able to measure different parameters such as forces, cells’ nanomechanical properties, or receptors mapping at the cell surface. AFM refines our understanding of microbes’ cell walls, mammals’ cell membranes, and the mechanism of drugs’ actions. AFM technologies are constantly improving. Certainly, in the coming years we will observe the increased use of AFM in studies with medical relevance and even more development in AFM technology such as FluidFM, AFM combined with other microscopy techniques, or maybe even an alternative to the presently used tools for patient diagnosis.
